# Effectiveness and Safety of Pyrotinib, and Association of Biomarker With Progression-Free Survival in Patients With HER2-Positive Metastatic Breast Cancer: A Real-World, Multicentre Analysis

**DOI:** 10.3389/fonc.2020.00811

**Published:** 2020-05-25

**Authors:** Qitong Chen, Dengjie Ouyang, Munawar Anwar, Ning Xie, Shouman Wang, Peizhi Fan, Liyuan Qian, Gannong Chen, Enxiang Zhou, Lei Guo, Xiaowen Gu, Boni Ding, Xiaohong Yang, Liping Liu, Chao Deng, Zhi Xiao, Jing Li, Yunqi Wang, Shan Zeng, Jinhui Hu, Wei Zhou, Bo Qiu, Zhongming Wang, Jie Weng, Mingwen Liu, Yi Li, Tiegang Tang, Jianguo Wang, Hui Zhang, Bin Dai, Wuping Tang, Tao Wu, Maoliang Xiao, Xiantao Li, Hailong Liu, Lai Li, Wenjun Yi, Quchang Ouyang

**Affiliations:** ^1^Department of General Surgery, The Second Xiangya Hospital, Central South University, Changsha, China; ^2^Department of General Surgery, Xiangya Hospital Central South University, Changsha, China; ^3^Department of Breast Internal Medicine, The Affiliated Cancer Hospital of Xiangya School of Medicine, Central South University, Changsha, China; ^4^Department of Breast Surgery, Xiangya Hospital, Central South University, Changsha, China; ^5^Department of Breast and Thyroid Surgery, Hunan Provincial People's Hospital, Changsha, China; ^6^Department of Breast and Thyroid Surgery, Central South University Third Xiangya Hospital, Changsha, China; ^7^Department of Oncology, The Affiliated Cancer Hospital of Xiangya School of Medicine, Cental South University, Changsha, China; ^8^Department of Oncology, The Second Xiangya Hospital, Central South University, Changsha, China; ^9^Department of Breast Medical Oncology, The Affiliated Cancer Hospital of Xiangya School of Medicine, Cental South University, Changsha, China; ^10^Department of Traditional Chinese Medicine, The Affiliated Cancer Hospital of Xiangya School of Medicine, Cental South University, Changsha, China; ^11^Department of Internal Medicine – Oncology, Xiangya Hospital Central South University, Changsha, China; ^12^Department of Breast Surgery, The First Hospital Hunan University of Chinese Medicine, Changsha, China; ^13^Department of Breast and Thyroid Surgery, The Affiliated ZhuZhou Hospital of Xiangya School of Medicine Central South University, Zhuzhou, China; ^14^Department of Oncology, The Affiliated ZhuZhou Hospital of Xiangya School of Medicine Central South University, Zhuzhou, China; ^15^Department of Breast Surgery, The Third People's Hospital of Yongzhou, Yongzhou, China; ^16^Department of Oncology, The First People's Hospital of Yueyang, Yueyang, China; ^17^Department of Breast and Thyroid Surgery, The First People's Hospital of Xiangtan City, Xiangtan, China; ^18^Department of Oncology, The Third People's Hospital of Changde, Changde, China; ^19^Department of Oncology, Xiangtan Central Hospital, Xiangtan, China; ^20^Department of General Surgery, Xiangtan Central Hospital, Xiangtan, China; ^21^Department of Oncology, Central Hospital of Shaoyang, Shaoyang, China; ^22^Department of Breast and Thyroid Surgery, Central Hospital of Shaoyang, Shaoyang, China; ^23^Department of Breast Surgery, Shaoyang Hospital of Traditional Chinese Medicine, Shaoyang, China; ^24^Department of Oncology, The First People's Hospital of Changde, Changde, China; ^25^Department of Oncology, The Third Hospital of Hunan University of Chinese Medicine, Zhuzhou, China; ^26^Department of Oncology, The Central Hospital of Yiyang, Yiyang, China; ^27^Department of Internal Medicine – Oncology, The First People's Hospital of Chenzhou, Chenzhou, China; ^28^Department of Breast and Thyroid Surgery, The People's Hospital of Xiangtan County, Xiangtan, China

**Keywords:** breast cancer, HER2, pyrotinib, tumor mutation burden, metastases

## Abstract

**Background:** Pyrotinib, an irreversible pan-ERBB inhibitor, has shown promising antitumour activity, and acceptable tolerability. This research was conducted to evaluate the actual use and effectiveness of pyrotinib in China, therefore, contributed to solve the problem of real-world data scarcity.

**Methods:** In this retrospective study, 168 patients who received pyrotinib treatment for HER2-positive metastatic breast cancer (MBC) in Hunan Province from June 2018 to August 2019 were included. Progression-free survival (PFS), tumor mutation burden (TMB), and drug-related adverse events (AEs) after pyrotinib administration were analyzed.

**Results:** The median PFS (mPFS) time in the 168 participants was 8.07 months. The mPFS times in patients with pyrotinib in second-line therapy (*n* = 65) and third-or-higher-line therapy (*n* = 94) were 8.10 months and 7.60 months, respectively. Patients with brain metastases achieved 8.80 months mPFS time. In patients with pyrotinib in third-or-higher-line therapy, patients who had previously used lapatinib still got efficacy but showed a shorter mPFS time (6.43 months) than patients who had not (8.37 months). TMB was measured in 28 patients, K-M curve (*P* = 0.0024) and Multivariate Cox analysis (*P* = 0.0176) showed a significant negative association between TMB and PFS. Diarrhea occurred in 98.2% of participants (in any grade) and 19.6% in grade 3–4 AEs.

**Conclusion:** Pyrotinib is highly beneficial to second-or-higher-line patients or HER2-positive MBC patients with brain metastases. Pyrotinib seems to be a feasible strategy both in combination of chemotherapeutic drugs or as a replacement of lapatinib if diseases progressed. TMB could be a potential predictor for evaluating pyrotinib's effectiveness in HER2-positive MBC.

## Introduction

Approximately 20–30% of patients with breast cancer demonstrate overexpression of human epidermal growth factor receptor 2 (HER2) (1). This type of breast cancer exhibits more-aggressive clinical behavior and poorer outcomes than those who do not overexpress HER2 (1). With the development of anti-HER2 therapies, such as trastuzumab (2), pertuzumab (3), lapatinib (4), neratinib (5), and ado-trastuzumab emtansine (T-DM1) (6), the prognoses of patients with HER2-positive breast cancer have improved significantly. However, resistance and AEs are frequently observed during HER2-directed therapy, and are obstacles to the continuous administration of these agents (7). Therefore, it is crucial to improve anti-HER2 strategies for patients who are intolerant of standard therapies, as well as determine the mechanisms of resistance.

Pyrotinib is an orally administered tyrosine-kinase inhibitor (TKI) that has been approved in China for the treatment of HER2-positive MBC (8). Preclinical data suggest that pyrotinib can irreversibly inhibit multiple receptor tyrosine kinases of the ERBB family (including HER1 [also known as epidermal growth factor receptor (EGFR)], HER2, and HER4), and effectively inhibit the proliferation of HER2-overexpressing cells both *in vivo* and *in vitro* (9, 10). Efforts are being made to evaluate the *in vivo* efficacy and safety of pyrotinib, and to determine the associated AEs. In a phase I pyrotinib-monotherapy study and a phase II pyrotinib-vs.-lapatinib study, the recommended dosage of oral pyrotinib was 400 mg once daily after a meal (11, 12). Whether monotherapy or combined therapy can lead to significantly improved objective response rates and PFS times with controllable toxicity (e.g., diarrhea) (11, 12). Although phase III clinical trials are in progressing, it cannot fully reflect the real-world treatment setting as there is lack of relevant data.

Besides real-world data to evaluate pyrotinib efficacy in the treatment of breast cancer, it is important to identify biomarkers to predict effectiveness of pyrotinib-based therapy. Although *PIK3CA* and *TP53* were found to be associated with low treatment efficacy of pyrotinib monotherapy in phase I study (11), this correlation was not observed in pyrotinib in combination with capecitabine therapy (13). Thus, these contrary results suggest that better indicators need to be explored to evaluate the efficacy of pyrotinib-based therapy. Currently, TMB is emerging as an outcome biomarker of immune checkpoint blockade response (14). The implication of TMB in other treatment settings, such as targeted therapy, is little unknown. Studies have shown that TMB can be used as a therapeutic marker of EGFR-TKI for lung cancer (15–17). Nevertheless, there are lack of researches focus on investigating the relationship between TMB and treatment outcomes in HER2-positive MBC, especially for pyrotinib-based treatments.

By analyzing real-world data from a multicentre study of patients with HER2-positive MBC who were treated with pyrotinib, this study aimed to evaluate the effects on PFS of the pyrotinib treatment line, the metastatic site, the use of pyrotinib in combination with other chemotherapeutic agents, and replacement of lapatinib. Simultaneously, the relationship between TMB and the outcome of pyrotinib treatment has been analyzed, in order to identify potential predictive or prognostic biomarkers for HER2-positive MBC. Finally, the AEs associated with pyrotinib treatment were also analyzed in this study.

## Patients and Methods

### Patient Eligibility and Study Design

The study used the following inclusion criteria: (i) eligible patients had a confirmed histological or cytological diagnosis of HER2-positive MBC (with tumor tissue protein expression demonstrated by immunohistochemistry [IHC] category 3+ or positive results of fluorescence *in situ* hybridization [FISH]); (ii) eligible patients had a measurable lesion as defined by the revised Response Evaluation Criteria in Solid Tumors guidelines version 1.1 (RECIST 1.1); (iii) eligible patients had adequate hematological, hepatic, and renal functions. No limits on the number of prior cytotoxic regimens for metastatic disease were set.

Patients were excluded if they discontinued pyrotinib treatment, either because of medication use in a neoadjuvant setting (*n* = 7), or for reasons unrelated to treatment progress [economic reasons [*n* = 27], severe AEs [*n* = 18]], or if they were lost to follow-up for other, unknown reasons (*n* = 12) (Supplementary Figure 1).

This study was a multicentre (*n* = 20), retrospective, real-world study (RWS) conducted from the Second Xiangya Hospital of Central South University (Hunan Province, China). Participants were women with MBC who started treatment with pyrotinib administered in standard clinical practice in one of the hospitals in Hunan Province. Patients received either 400 mg pyrotinib (*n* = 153, 91.1%) or 320 mg pyrotinib (*n* = 15, 8.9%) once daily, in 21-day cycles, in addition to other medication as indicated in Table 1. All patients provided their written informed consent, and the study was reviewed and approved by the Research Ethics Committee of the Second Xiangya Hospital of Central South University.

**Table 1:** Characteristics of 168 patients with HER2-positive MBC.

	**Total**	**%**	**Median PFS (95% CI)**
	**(*n* = 168)**		
**Median age (range), years**	50 (28–73)		
<50	82	48.81	7.73 (6.624–8.836)
≥50	86	51.19	8.67 (7.242–10.098)
**ECOG scale**			
0-1	155	92.26	8.17 (7.149–9.191)
≥2	13	77.38	7.03 (5.917–8.143)
**Menopausal status**			
Postmenopausal	93	55.36	7.47 (6.723–8.217)
Premenopausal	75	44.64	8.97 (7.739–10.201)
**Hormone-receptor status**			
Positive	90	53.57	8.00 (6.468–9.532)
Negative	72	42.86	8.67 (7.269–10.071)
Unknown	6	3.57	5.80 (0.000–12.113)
**Treatment stage**			
First-line	9	5.36	-
Second-line	65	38.69	8.17 (6.466–9.874)
Third-or-higher-line	94	55.95	7.60 (6.352–8.848)
**Treatment type**			
No previous use of capecitabine			
Pyrotinib + capecitabine	114	67.86	8.67 (7.110–10.230)
Previous use of capecitabine			
Pyrotinib + abraxane	19	11.31	8.70 (4.405–12.995)
Pyrotinib + trastuzumab	12	7.14	5.20 (0.000–10.683)
Pyroti nib + others	23	13.69	6.67 (5.653–7.687)
**Metastatic site**			
Soft tissue and/or bone	30	17.86	6.70 (5.145–8.255)
Organ	99	58.93	8.07 (6.741–9.399)
Brain	39	23.21	8.80 (6.588–11.012)

*ECOG, Eastern Cooperative Oncology Group; PFS, progression-free survival; CI, confidence interval*.

### Data Collection

Data collection was performed retrospectively by two trained staff using a standardized data-collection method from the routine clinical information system, and were documented in an electronic case-report form. Data were monitored using automated plausibility checks and on-site monitoring.

First-line treatment was defined as treatment for a patient with *de novo* stage IV breast cancer not treated previously with anti-HER2 medications, or for a patient with recurrence >12 months after discontinuation of trastuzumab. Second-line treatment was that given to a patient with recurrence within 12 months of discontinuation of trastuzumab, or recurrence during adjuvant therapy with trastuzumab, or progression following first-line treatment. Third-or-higher-line treatment was that given to a patient with progression or recurrence following second-line treatment and for which any one of the anti-HER2 drugs or chemotherapeutic drugs had been changed.

AEs that were recorded considered at least possibly pyrotinib-treatment-related by the treating physician. The date of onset of AEs and their severity according to the National Cancer Institute Common Terminology Criteria for Adverse Events (version 4.0) were recorded.

### Definition of HER2 Status and Grading

Information about the HER2 status, and grading were obtained for documentation purposes for each tumor that had been biopsied. Samples for these analyses originated from primary tumors, local recurrences, and metastatic sites. The biomarker status was determined for HER2 as follows: if a biomarker assessment of the metastatic site was available, this receptor status was used. If no information was available for metastases, the latest biomarker results from the primary tumor were used. All patients who had ever received anti-HER2 therapy were assumed to be HER2 positive. A positive HER2 status required an IHC score of 3+ or positive FISH results according to the ASCO/CAP 2018 HER2 test guideline (18).

### Assessment of TMB

In our cohort, 28 out of 168 peripheral-blood samples collected (only 28 patients consent) prior to pyrotinib treatment were subjected to a next-generation sequencing (NGS) assay (OncoMD/OncoMD Plus [Beijing, China], comprising a customized panel of 1,021 genes) at Geneplus-Beijing Institute (http://www.geneplus.org.cn), Beijing, China. The TMB was determined by integrated mutation profiling testing of actionable cancer targets within the same gene panel. TMB [mutations per megabase [mut/Mb]] was calculated from sequenced DNA (19).

### End Point and Assessments

The primary end point was PFS, which was defined as the time from drug administration to death or disease progression (whichever came first), as assessed by the investigator, according to RECIST 1.1 criteria. The end point was analyzed in the full population of all 168 patients.

Six patients were censored at the last valid disease-assessment date or at the last prior assessment after two missed visits. Clinical follow-up was scheduled every 2–3 weeks during treatment. Imaging follow-up was scheduled every 1–2 months, according to the standard clinical practice.

### Statistical Analyses

Pearson's χ^2^ test or Fisher's exact test were used for comparisons of categorical variables in different groups of patients. Continuous distributions which did not follow a normal distribution were compared using a non-parametric Mann–Whitney *U*-test. Kaplan–Meier estimates of PFS were compared between treatment arms using a log-rank test. Median survival times and 95% confidence intervals (CI) were estimated. Univariable and multivariable Cox proportional hazard models were used for assessment of the adjusted effects of covariates on PFS. Statistical analyses were performed using SPSS statistical software version 22.0 (SPSS, NY, USA: IBM Corporation), R 3.6.3 and Prism analysis and graphing software version 8.0.1 (GraphPad, San Diego, USA). A two-sided *P* < 0.05 was considered statistically significant.

## Results

### Baseline Characteristics and Patients' Outcomes

Between June 2018 and August 2019, we enrolled 168 patients with HER2-positive MBC. Among these patients, the median age was 50 years (range 28–73 years). The specific baseline characteristics, mPFS times, and 95% CIs associated with particular ranges are presented in Table 1.

**Figure 1:**
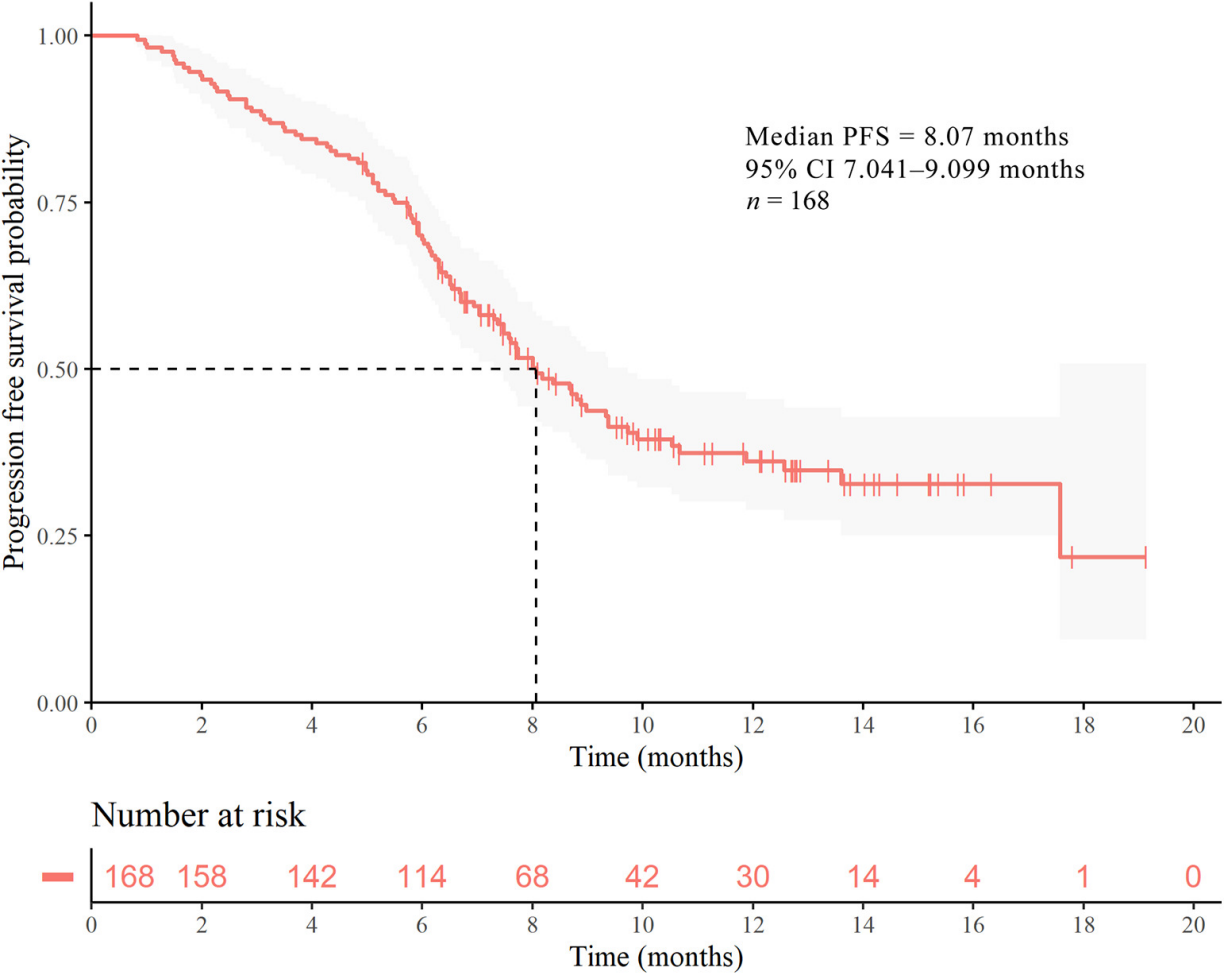
Kaplan–Meier curves of PFS for patients with HER2-positive MBC. Survival analysis for the entire cohort (*n* = 168).

**Figure 2:**
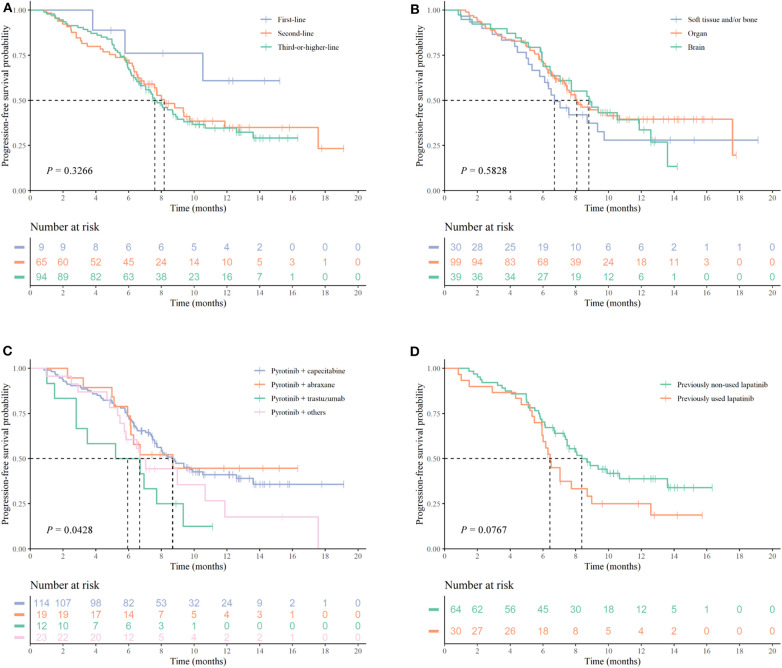
Kaplan–Meier curves of PFS for patients with HER2-positive MBC. **(A)** Survival analysis comparing first-line (*n* = 9), second-line (*n* = 66), and third-or-higher-line (*n* = 93) pyrotinib-containing treatments. **(B)** Survival analysis according to metastatic sites. **(C)** Survival analysis according to treatment regimens. **(D)** Survival analysis according to previously used lapatinib (*n* = 30) or not (*n* = 64). *P-*values are from univariate log-rank tests.

Following up to January 2020, the median follow-up time was 7.30 months. The mPFS time in the study population was 8.07 months (95% CI 7.041–9.099 months) (Figure 1). The number of PFS events was 99 (58.9%). The mPFS time in patients with second-line pyrotinib treatment was 8.17 months, and the PFS for third-or-higher-line treatment was 7.60 months. There were too few progression events in the group of nine patients with first-line treatment to enable calculation of the mPFS. The mPFS time was shorter for third-or-higher-line pyrotinib treatment than for first-line and second-line treatment (*P* = 0.3266, the difference is not significant, Figure 2A). Eighteen patients (10.71%) achieved complete response (CR) and 50 (29.76%) achieved partial response (PR) for an objective response rate (ORR) of 40.47% (95% CI, 22.7–54.2%).

Log-rank test indicated that age (*P* = 0.9137), hormone-receptor status (*P* = 0.7251), and classification of metastatic sites (*P* = 0.5828, Figure 2B) at the time of pyrotinib initiation had no significant associations with PFS. The mPFS time in patients (*n* = 39, 23.21%) with brain metastases, including those who also had metastases to other organs and those with local metastases, was 8.80 months. the mPFS for patients (*n* = 99, 58.93%) with organs metastases was 8.07 months and patients (*n* = 30, 17.86%) with soft tissue and/or bone metastases was 6.70 months. In patients treated with the combination of pyrotinib (pyrotinib with capecitabine, abraxane, trastuzumab, vinorelbine, etoposide, or S-1), our results suggested a significant difference PFS times (*P* = 0.0428, Figure 2C). The PFS time of the pyrotinib combined with capecitabine group was 8.67 months while referring to patients who previously used capecitabine, pyrotinib combined with abraxane was 8.70 months, pyrotinib with trastuzumab was 5.20 months, and pyrotinib combined with other drugs was 6.67 months.

### Effectiveness of Switching Use of TKIs

In patients with anti-her2 treatment of third-or-higher-line treatment, patients who had previously used lapatinib (*n* = 30 mPFS: 6.43 months 95% CI 5.883–6.977 months) showed a different (*P* = 0.0767, Figure 2D) PFS than patients who had not used lapatinib (*n* = 64 mPFS: 8.37 months 95% CI 6.402–10.338 months). There was no statistical difference in the baseline data between the two groups (Supplementary Table 1).

### High TMB Is Associated With Poor PFS

**Figure 3:**
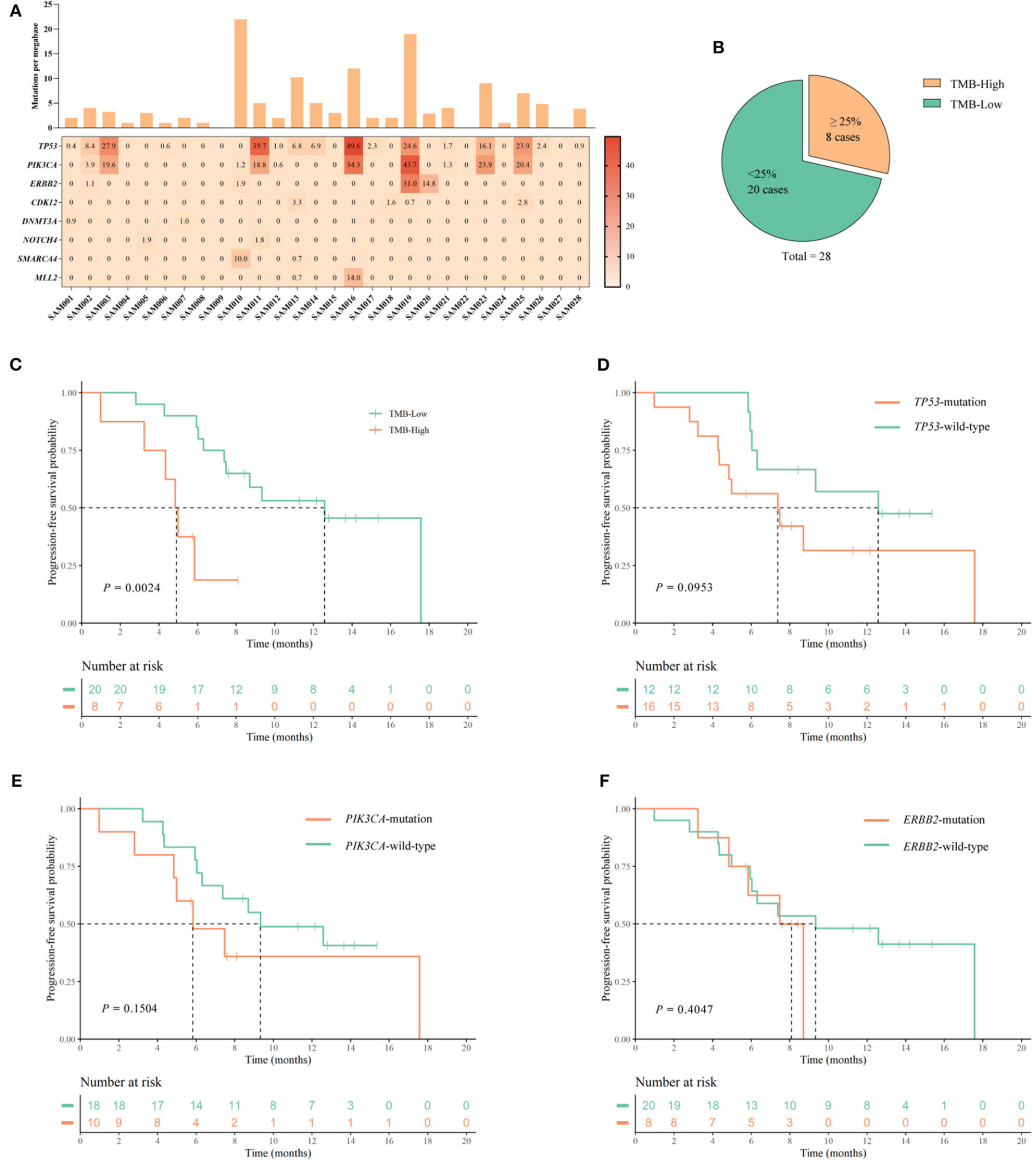
Assessment of the TMB in ctDNA, and analysis of PFS associated with mutations in specific genes. **(A)** The frequencies (≥2) of mutated genes in 28 samples are plotted as a heatmap, and the TMB (mutations per megabase) of each ctDNA sample is shown in a histogram. **(B)** Classification of patients into TMB-High, and TMB-Low groups containing ≥25% highest TMB (≥5.0 mut/Mb), and <25% highest TMB (<5.0 mut/Mb), respectively, of the 28 patients. **(C–F)** Kaplan–Meier curves for PFS according to: **(C)** TMB status; **(D)** *TP53* mutation status; **(E)** *PI3KCA* mutation status; and **(F)** *ERBB2* mutation status. *P-*values are from univariate log-rank tests.

Next, we assessed the potential of tumor mutations detected in ctDNA as predictive biomarkers of pyrotinib effectiveness. Blood samples were available from 28 out of 168 patients, and NGS was performed on ctDNA. Detection of ctDNA mutations is summarized in Figures 3A,B. Among 28 patients, 21 (75.0%) had a ctDNA TMB of >1 mut/Mb, and the median TMB per patient was 3 mut/Mb (range 0–22 mut/Mb, mean = 4.71 mut/Mb). The ctDNA TMB was >3 mut/Mb in 13 patients (46.4%), and ≥6 mut/Mb in six patients (21.4%).

The 28 patients were assigned to the following TMB categories: the eight patients (≥25%, ≥5.0 mut/Mb) with the highest TMB were classified as high TMB (TMB-H), and 20 patients (<25%, <5.0 mut/Mb) were low TMB (TMB-L) like other studies (20) (Figure 3B). There were no statistical differences in baseline characteristics between the high and low TMB groups (Supplementary Table 2). We compared the Kaplan–Meier PFS curves associated with these two TMB categories, and found a significant difference according to the log-rank test (*P* = 0.0024) (Figure 3C). mPFS values were 12.57 months (95% CI 8.998–16.142) for TMB-L and 4.83 months (95% CI 3.943–5.717 months) for TMB-H. These data suggest that high TMB may be a prognostic marker for poor PFS in patients undergoing HER2-directed treatments and chemotherapy. In comparisons of Kaplan–Meier PFS curves associated with the presence or absence of mutations in the individual *TP53* (*n* = 16), *PIK3CA* (*n* = 10), and *ERBB2* (*n* = 8) genes, no significant differences were identified by log-rank tests (Figures 3D–F).

Univariate Cox analysis revealed that only TMB categorization (*P* = 0.0054) was significantly associated with PFS. Multivariable Cox regression analyses showed that metastatic site (*P* = 0.0498), treatment stage (*P* = 0.0343), and TMB status (*P* = 0.0176) might be associated with PFS. Results of univariate and multivariate Cox analysis were presented in Table 2.

**Table 2:** Univariable and multivariable analysis of factors for prediction of PFS in 28 patients with HER2-positive metastatic breast cancer who had NGS analysis.

**Characteristics**	**Univariable cox**		**Multivariable cox**	
	**HR (95% CI)**	***P*-value**	**HR (95% CI)**	***P*-value**
Age	0.9736 (0.3499–2.7089)	0.9592	0.1299 (0.0085–1.9665)	0.1409
Menopausal_status	1.3921 (0.5132–3.7760)	0.5158	3.2431 (0.2570–40.921)	0.3630
Hormone-receptor status	0.5628 (0.2249–1.4085)	0.2194	0.6746 (0.1688–2.6959)	0.5776
Metastatic site	0.6648 (0.3187–1.3869)	0.2766	0.3254 (0.1059–0.9993)	**0.0498**
Drug line	1.3421 (0.5342–3.3715)	0.5312	3.8687 (1.1048–13.546)	**0.0343**
Durg combination	1.2336 (0.8342–1.8241)	0.2927	1.3745 (0.7464–2.5312)	0.3070
TMB	5.2778 (1.6332–17.055)	**0.0054**	13.547 (1.5752–116.50)	**0.0176**
*TP53*	2.1558 (0.7610–6.1067)	0.1481	1.0838 (0.2226–5.2767)	0.9206
*PIK3CA*	1.4426 (0.4782–4.3514)	0.5153	0.9786 (0.2461–3.8900)	0.9755
*ERBB2*	1.7936 (0.6352–5.0642)	0.2699	1.1438 (0.1457–8.9753)	0.8982

*NGS, next-generation sequencing; PFS, progression-free survival; HR, hazard ratio; CI, confidence interval; TMB, tumor mutation burden. The bold values means reaching the significance of statistics*.

### AEs

Diarrhea was the most common AE observed in our cohort, occurring in 98.7% of participants. Diarrhea was mainly grade 1–2, but in 19.6% of patients was grade 3–4 (Table 3). Apart from diarrhea, the most frequent grade 3–4 AEs were nausea and vomiting (in 7.7% of patients), leukopenia (in 7.7% of patients), and hand–foot syndrome (in 6.5% of patients) (Table 3).

**Table 3:** Pyrotinib-related AEs of all grades and grade 3–4.

	**Pyrotinib dose 320 mg daily (*****n*** **=** **15)**		**Pyrotinib dose 400 mg daily (*****n*** **=** **153)**		**Total (*****n*** **=** **168)**	
**AE**	**All grade**	**Grade 3–4**	**All grade**	**Grade 3–4**	**All grade**	**Grade 3–4**
Diarrhea	14 (93.0%)	3 (20.0%)	151 (98.7%)	31 (20.2%)	165 (98.2%)	33 (19.6%)
Nausea & vomiting	8 (53.3%)	1 (6.7%)	76 (49.7%)	12 (7.8%)	84 (50.0%)	13 (7.7%)
Leukopenia	7 (46.7%)	1 (6.7%)	75 (49.0%)	12 (7.8%)	82 (48.8%)	13 (7.7%)
Decreased appetite	6 (40%)		54 (35.3%)	4 (2.6%)	60 (35.7%)	4 (2.4%)
Hand-foot syndrome	4 (26.7%)	1 (6.7%)	43 (28.1)	10 (6.5%)	47 (28.0%)	11 (6.5%)
Asthenia	4 (26.7%)		28 (18.3%)		32 (19%)	
Oral ulceration	2 (13.3%)		17 (11.1%)	3 (2%)	19 (11.3%)	3 (1.8%)
Rash	1 (6.7%)		13 (8.5%)		14 (8.3%)	
Dizziness	0 (0%)		9 (5.9%)		9 (5.4%)	
Muscle spasms	1 (6.7%)		5 (3.4%)		6 (3.6%)	
Paronychia	1 (6.7%)		5 (3.3%)	2 (1.3%)	6 (3.6%)	2 (1.2%)
Cough	2 (13.3%)		4 (2.6%)		6 (3.6%)	
Haematochezia	0 (0%)		3 (2%)		3 (1.8%)	
Abdominal Pain	0 (0%)		3 (2%)		3 (1.8%)	
Oedema	0 (0%)		3 (2%)		3 (1.8%)	
Epistaxis	1 (6.7%)		1 (0.7%)		2 (1.2%)	

*Pyrotinib-related adverse events (AEs) includes definitely related AEs and probably related AEs*.

### Discussion

In this study, pyrotinib was orally administered to patients with HER2-positive MBC. The study reached its primary end point. Generally, the result of data analysis demonstrates that pyrotinib treatment led to a median survival time of 8.07 months. This real-world research is a crucial complement to current clinical trials of pyrotinib.

Currently, the international recommended treatment regimen for HER2-positive MBC is trastuzumab plus pertuzumab with docetaxel; the second choice is TDM1, lapatinib or pertuzumab plus chemotherapy (21). Due to the diverse limitations of drug use in China, the preferred treatment is trastuzumab with docetaxel, and the fall-back plans include pyrotinib or lapatinib combined with capecitabine or trastuzumab overline therapy (21). Evidence suggests that in the second-line treatment regimen, the strategy of trastuzumab plus pertuzumab combined with capecitabine chemotherapy maximizes PFS to 11.1 months (22). By comparison, T-DM1 (6) and lapatinib in combination with capecitabine (4), have demonstrated PFS of 9.6 and 8.4 months, respectively. In this study, the PFS for patients with HER2-positive MBC was observed to be 8.17 months and 7.60 months for second-line and third-or-higher-line pyrotinib treatments separately. Pyrotinib acts directly on the intracellular tyrosine-kinase domain, which is unlike traditional anti-HER2 therapy, and can completely block the downstream pathway activation that results from ligand binding to ERBB-family homodimers and heterodimers (10). This irreversible binding mode enhances the effects of pyrotinib and it also suggests drug resistance associated with HER2 over-expression may be overcame by pyrotinib (23). The results perform that pyrotinib could offer a breakthrough treatment in a second-or-higher-line treatment setting to some extent.

Brain metastases frequently occur in breast cancer, especially in HER2-positive MBC, for which the rate of brain metastasis is reportedly as high as 20–50% (24). Continuous anti-HER2 treatment after brain metastasis in HER2-positive breast cancer can reduce the risk of death from extracranial metastases by ~50% (25). Trastuzumab, as a macromolecular monoclonal antibody, is not easy to cross the blood-brain barrier (BBB) (26). TKIs such as lapatinib performed a higher rate on crossing the BBB to enter the brain than trastuzumab, together with higher concentration surrounded in the brain metastases. (27). In clinical trials, lapatinib (28) or neratinib plus capecitabine (29) have resulted in PFS of 4.6 and 5.5 months in patients with brain metastases. T-DM1 has led to PFS of 5.9 months (30). In the present study, we found PFS of 8.80 months with pyrotinib in patients with brain metastases. Consequently, as a novel small molecule TKI, pyrotinib is significantly to improve the prognosis of patients with brain metastasis, although it requires further study for investigating the mechanisms of entering BBB and effectiveness of patients with brain metastasis.

Results from PHENIX study showed that the PFS time of pyrotinib combined with capecitabine could reach 11.1 months, together with an optimal PFS of 6.9 months for patients with brain metastasis (31). The differences existed between PHENIX study and this study might be caused by different population sample size and cohort. In PHENIX study, 79.5% patients were visceral metastasis; patients with brain metastases or with third-or-higher treatment lines accounted for 11.4 and 8.8% separately (31), while these patients accounted for a higher proportion in present study. Furthermore, the effectiveness of pyrotinib combined with other chemotherapeutic drugs in patients who had previously used capecitabine was compared with pyrotinib plus capecitabine in this study. The result indicated that the treatment strategy of pyrotinib plus abraxane has no statistical difference from pyrotinib plus capecitabine, while it is superior to the combination of pyrotinib plus trastuzumab or other chemotherapy. In other words, if disease progressed in those HER2-positive MBC patients treated by capecitabine, it is possible to choose other chemotherapy drugs to combine with pyrotinib.

There is a lack of research on switching utilization of TKIs. PHENIX study excluded patients who were treated with lapatinib (31). Present study explored the switching use of TKIs anti-HER2 therapy, the result illustrates that taking pyrotinib after the initial use of lapatinib in third-or-higher treatment lines still got efficacy. Hence, it suggests that TKIs replacement could also be beneficial to patients while further investigations are still required.

Previous studies of the predictive values of tumor mutations have been based on analyses of primary tumor samples (32, 33). However, because of intratumour heterogeneity of gene profiles, biopsies of the primary tumor cannot reflect the whole picture of gene mutation in the patient, especially in individuals with different mutation status in the primary and metastatic tumors (34, 35). ctDNA has been suggested as an alternative to tumor biopsy samples for mutational analysis (36). Genetic analysis in HER2-positive MBC should take account of the multiple signal-transduction pathways [especially the activation of PI3K/Akt/mTOR pathway (37, 38) and ERBB2 mutation (23)] that are associated with resistance to anti-HER2 therapy. In addition to individual mutations, this study evaluated the overall TMB as a predictor of pyrotinib effectiveness. Previous study showed that high TMB has been identified as a prognostic marker for good overall survival in patients with HER2-positive MBC who undergo conventional HER2-directed treatments and chemotherapy (39). In the current study, high TMB was associated with poor PFS under pyrotinib-based treatment. This conversely relationship between TMB and PFS might be related to sample and antibody-dependent cells mediate cytotoxic effects of trastuzumab and pertuzumab. To the best of our knowledge, our study is the first to determine the association between ctDNA TMB and PFS in patients receiving pyrotinib therapy.

The incidence of pyrotinib-related AEs that we observed, such as diarrhea, nausea, vomiting et al. were similar with previous studies (12, 13, 31). Diarrhea is the most common AE observed in present study. All pyrotinib-related AEs were effectively controlled with treatment and did not lead to discontinuation of pyrotinib treatment during the study. Notably, leukopenia was present in 49% of patients who received 400 mg pyrotinib doses in our study, which was a higher incidence than that in a previous phase II study (46.2%), probably because 32.1% of the patients included in our study were being treated in combination with other chemotherapeutic drugs.

The limitations of this study are the non-selectivity of the sample cohort in RWS and fewer patients in this study were previously treated with T-DM1 and pertuzumab. Therefore, the results of some studies are required for further verified by clinical randomized controlled studies.

In conclusion, this RWS contributed to validate the efficacy and safety of pyrotinib in advanced HER2-positive breast cancer. It brings benefits for second-or-higher-line patients, as well as provide more possible strategies for those with brain metastases. In addition to the current regimen of pyrotinib combined with capecitabine, pyrotinib can also be combined with other chemotherapeutic drugs, and the TKIs switching therapy is also beneficial to targeted-group patients. Furthermore, in this study, TMB is identified to be a possible candidate biomarker for prediction of the response to pyrotinib-based therapy, which is desired to be further investigated in next steps.

## Data Availability Statement

The datasets generated for this study are available on request to the corresponding author.

## Ethics Statement

All patients provided their written informed consent, and the study was reviewed and approved by the Research Ethics Committee of the Second Xiangya Hospital.

## Author Contributions

MA and QC collected and compiled the patients' information. QC and DO performed the literature search, data extraction and statistical analysis, and drafted the manuscript. WY and QO designed and supervised the study. Other authors conducted the clinical therapy of patients, followed up with the patients' information, and assess and recorded the AEs. All authors have read and approved the final manuscript.

## Conflict of Interest

The authors declare that the research was conducted in the absence of any commercial or financial relationships that could be construed as a potential conflict of interest.

## Supplementary Material

Supplementary Figure 1 Flow chart of the selection process for patients with HER-2-positive MBC who underwent pyrotinib treatment.

Supplementary Table 1 The characteristic information of patients in pyrotinib third-or-higher-line therapy who have previously used lapatinib or not.

Supplementary Table 2 Characteristics of 28 patients with HER2-positive metastatic breast cancer, according to TMB categorisation.

## Abbreviations

AEs: Adverse events
BBB: Blood-brain barrier
*CDK12*: Cyclin Dependent Kinase 12
CI: Confidence intervals
ctDNA: Circulating tumor DNA
*DNMT3A*: DNA Methyltransferase 3 Alpha
EGFR: Epidermal growth factor receptor
ECOG: Eastern Cooperative Oncology Group
ERBB: Erb-B2 Receptor Tyrosine Kinase
ER: Estrogen receptor
RECIST: Evaluation criteria in solid tumors
FISH: Fluorescence *in situ* hybridization
HER2: Human epidermal growth factor receptor 2
IHC: Immunohistochemistry
MBC: Metastatic breast cancer
NGS: Next-generation sequencing
*NOTCH4*: Notch Receptor 4
PFS: Progression-free survival
*PIK3CA*: Phosphatidylinositol-4,5-Bisphosphate 3-Kinase Catalytic Subunit Alpha
PR: Progesterone receptor
S-1: Tegafur, gimeracil and oteracil Potassium
*SMARCA4*: SWI/SNF related, matrix associated, Actin dependent regulator of Chromatin, subfamily A, member 4
*MLL2*: Lysine methyltransferase 2D
T-DM1: Ado-trastuzumab emtansine
TKI: Tyrosine-kinase inhibitor
TMB: Tumor mutation burden
*TP53*: Tumor protein P53
mPFS: Median progression-free survival.
