# Corrigendum: Effectiveness and Safety of Pyrotinib, and Association of Biomarker With Progression-Free Survival in Patients With HER2-Positive Metastatic Breast Cancer: A Real-World, Multicentre Analysis

**DOI:** 10.3389/fonc.2021.661128

**Published:** 2021-03-05

**Authors:** Qitong Chen, Dengjie Ouyang, Munawar Anwar, Ning Xie, Shouman Wang, Peizhi Fan, Liyuan Qian, Gannong Chen, Enxiang Zhou, Lei Guo, Xiaowen Gu, Boni Ding, Xiaohong Yang, Liping Liu, Chao Deng, Zhi Xiao, Jing Li, Yunqi Wang, Shan Zeng, Jinhui Hu, Wei Zhou, Bo Qiu, Zhongming Wang, Jie Weng, Mingwen Liu, Yi Li, Tiegang Tang, Jianguo Wang, Hui Zhang, Bin Dai, Wuping Tang, Tao Wu, Maoliang Xiao, Xiantao Li, Hailong Liu, Lai Li, Wenjun Yi, Quchang Ouyang

**Affiliations:** ^1^Department of General Surgery, The Second Xiangya Hospital, Central South University, Changsha, China; ^2^Department of General Surgery, Xiangya Hospital Central South University, Changsha, China; ^3^Department of Breast Internal Medicine, The Affiliated Cancer Hospital of Xiangya School of Medicine, Central South University, Changsha, China; ^4^Department of Breast Surgery, Xiangya Hospital, Central South University, Changsha, China; ^5^Department of Breast and Thyroid Surgery, Hunan Provincial People's Hospital, Changsha, China; ^6^Department of Breast and Thyroid Surgery, Central South University Third Xiangya Hospital, Changsha, China; ^7^Department of Oncology, The Affiliated Cancer Hospital of Xiangya School of Medicine, Cental South University, Changsha, China; ^8^Department of Oncology, The Second Xiangya Hospital, Central South University, Changsha, China; ^9^Department of Breast Medical Oncology, The Affiliated Cancer Hospital of Xiangya School of Medicine, Cental South University, Changsha, China; ^10^Department of Traditional Chinese Medicine, The Affiliated Cancer Hospital of Xiangya School of Medicine, Cental South University, Changsha, China; ^11^Department of Internal Medicine – Oncology, Xiangya Hospital Central South University, Changsha, China; ^12^Department of Breast Surgery, The First Hospital Hunan University of Chinese Medicine, Changsha, China; ^13^Department of Breast and Thyroid Surgery, The Affiliated ZhuZhou Hospital of Xiangya School of Medicine Central South University, Zhuzhou, China; ^14^Department of Oncology, The Affiliated ZhuZhou Hospital of Xiangya School of Medicine Central South University, Zhuzhou, China; ^15^Department of Breast Surgery, The Third People's Hospital of Yongzhou, Yongzhou, China; ^16^Department of Oncology, The First People's Hospital of Yueyang, Yueyang, China; ^17^Department of Breast and Thyroid Surgery, The First People's Hospital of Xiangtan City, Xiangtan, China; ^18^Department of Oncology, The Third People's Hospital of Changde, Changde, China; ^19^Department of Oncology, Xiangtan Central Hospital, Xiangtan, China; ^20^Department of General Surgery, Xiangtan Central Hospital, Xiangtan, China; ^21^Department of Oncology, Central Hospital of Shaoyang, Shaoyang, China; ^22^Department of Breast and Thyroid Surgery, Central Hospital of Shaoyang, Shaoyang, China; ^23^Department of Breast Surgery, Shaoyang Hospital of Traditional Chinese Medicine, Shaoyang, China; ^24^Department of Oncology, The First People's Hospital of Changde, Changde, China; ^25^Department of Oncology, The Third Hospital of Hunan University of Chinese Medicine, Zhuzhou, China; ^26^Department of Oncology, The Central Hospital of Yiyang, Yiyang, China; ^27^Department of Internal Medicine – Oncology, The First People's Hospital of Chenzhou, Chenzhou, China; ^28^Department of Breast and Thyroid Surgery, The People's Hospital of Xiangtan County, Xiangtan, China

**Keywords:** breast cancer, HER2, pyrotinib, tumor mutation burden, metastases

In the original article, there was an error. The following line was incorrect: “Second-line treatment was that given to a patient with recurrence within 12 months of discontinuation of trastuzumab, or recurrence during adjuvant therapy with trastuzumab.”

A correction has been made to *Patients and Methods, Data Collection*, paragraph 2:

“Second-line treatment was that given to a patient with recurrence within 12 months of discontinuation of trastuzumab, or recurrence during adjuvant therapy with trastuzumab, or progression following first-line treatment.”

In the original article, the second subtitle of the *Results* section was also incorrect: “Effeteness of Switching Use of TKIs”

The correct version of this subtitle is as follows: “Effectiveness of Switching Use of TKIs”

The authors apologize for these errors and state that they do not change the scientific conclusions of the article in any way. The original article has been updated.

