# Supplementary figures and images for: Effectiveness and Safety of Pyrotinib, and Association of Biomarker With Progression-Free Survival in Patients With HER2-Positive Metastatic Breast Cancer: A Real-World, Multicentre Analysis
Source: Front Oncol. 2020 May 25;10:811. doi: 10.3389/fonc.2020.00811 (PMC7263174; doi:10.3389/fonc.2020.00811)

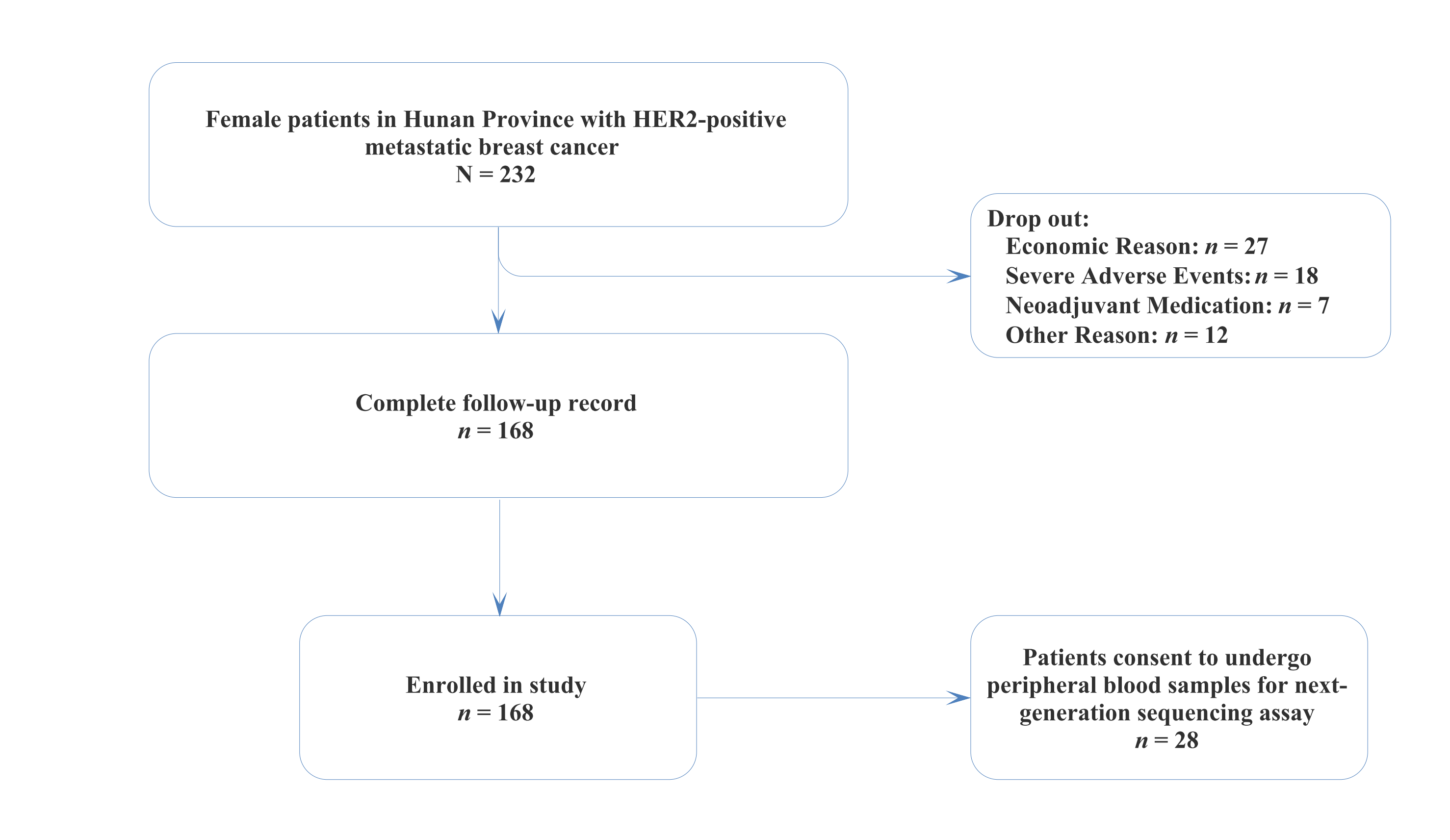

Supplement: Supplementary Figure 1 — Flow chart of the selection process for patients with HER-2-positive MBC who underwent pyrotinib treatment. [file Image_1.TIF]
